# Effects of Single-Nucleotide Polymorphisms in Calmodulin-Dependent Protein Kinase Kinase 2 (CAMKK2): A Comprehensive Study

**DOI:** 10.1155/2020/7419512

**Published:** 2020-10-06

**Authors:** Zoya Khalid, Omar Almaghrabi

**Affiliations:** ^1^Computational Biology Research Lab, Department of Computer Science, National University of Computing and Emerging Sciences (NUCES-FAST), Islamabad, Pakistan; ^2^Department of Biology, College of Science, University of Jeddah, Saudi Arabia

## Abstract

Calmodulin-dependent protein kinase kinase 2 (CAMKK2) is a protein kinase that belongs to the serine/threonine kinase family. It phosphorylates kinases like CAMK1, CAMK2, and AMP, and this signaling cascade is involved in various biological processes including cell proliferation, apoptosis, and proliferation. Also, the CAMKK2 signaling activity is required for the healthy activity of the brain which otherwise can cause diseases like bipolar disorders and anxiety. The current study is based on *in silico* bioinformatics analysis that combines sequence- and structure-based predictions to mark a SNP as damaging or neutral. The combined results from sequence-based, evolutionary conservation-based, and consensus-based tools have predicted a total of 18 nsSNPs as deleterious, and these nsSNPs were further subjected to structure-based analysis. The six mutant models of V195A, V249M, R311C, F366Y, P389T, and W445C showed a higher deviation from the wildtype protein model and hence were further taken for docking studies. The molecular docking analysis has predicted that these mutations will also be disruptive to the protein-protein interactions between CAMKK2 and PRKAG1 which will create an evident reduction in the kinase activity. The current study has enlightened us that a few of the significant mutations are prime candidates in CAMKK2 which could be the fundamental cause of various bipolar and psychiatric disorders. This is the first detailed study that predicts the deleterious nsSNPs in CAMKK2 and contributes positively in providing a better understanding of disease mechanisms.

## 1. Introduction

Calcium ions (Ca^2+^) are secondary messengers with an important regulatory role in cell signaling including hormone signaling, cell cycle regulation, and gene expression. Calmodulin (CaM), a small protein with a length of 148 amino acids, belongs to four EF-hand motifs, and each one of the four EF-hand motifs binds to Ca^2+^ ions. When calmodulin binds with Ca^2+^, it introduces conformational changes both locally by changing the EF-hand motifs and globally by increasing the alpha helix percentage in the protein [1]. One of the major functions of CaM is to activate the family of protein kinases which are CaM-dependent protein kinases or cited as CaM kinases. Among them, one of the CaM kinases is CAMKK2, which belongs to the serine/threonine kinase family. It phosphorylates various kinases like CAMK1, CAMK4, and AMP-activated protein kinases (AMPK). The signaling pathway of CAMKK2 is involved in various biological processes which include cell proliferation, energy balance, homeostasis, and apoptosis [2]. The human CaMKK2 locus has a total length of 40 kb pairs, and it is located at chromosome 12q24.2 and contains 18 exons and 17 introns. Polyadenylation of the last two exons generates two transcripts that play a part in neuronal differentiation. CAMKK2 activates AMPK by phosphorylating it at residue Thr172. The kinase has a catalytic alpha subunit which is encoded by PRKAA1 and PRKAA2, while the regulatory subunit is encoded by PRKAG1 and PRKAG2. These two kinases together make a stable multiprotein complex which is further regulated by the presence of Ca^2+^ ions [3].

Single-nucleotide polymorphisms (SNPs) are the most common type of genetic variation, and identifying those SNPs which have a phenotypic effect is a major challenge for medical researchers. SNPs more frequently occur in the noncoding regions which include the 5′UTR, 3′UTR, and intronic regions. The SNPs occurring in the protein-coding regions are also called as nonsynonymous SNPs (nsSNPs) that alter the encoded amino acid and cause structural and functional changes in the protein. Those SNPs that cause structural-functional damage are termed as deleterious SNPs and can further be associated with disease formation [4].

CAMKK2 is highly expressed in the nervous system and hence is involved in providing neuronal plasticity and also regulating axonal growth and dendrite formation. The CAMKK2 activity is important for normal and healthy brain development. The upregulation of CAMKK2 is associated with hepatic cancer and prostate cancer, while the downregulation of CAMKK2 is associated with human schizophrenia and bipolar disorders. Lately, numerous studies have been conducted to analyze the effects of nsSNPs on the structure and function of the protein. One study reported nine point mutations that are occurring at the phosphorylation site of CAMKK2 and hence decrease the autonomous activity of the protein [5]. The study carried out by O'Brien et al. determines the effect of T85S substitution, which is an autophosphorylation site on the activity of CAMKK2 and is also associated with behavioral disorders including anxiety. The autophosphorylation occurring at this position decreases the CAMKK2 activity and hence causes neuronal disorders [6]. It has been experimentally determined by Ling et al. that the R311C variant occurring in the loop region of the catalytic domain of CAMKK2 affects kinase activity [7]. This variant abolishes the T85 autophosphorylation site and creates a negative effect on the wildtype CAMKK2. The mutation is also predicted to be one of the genetic causes of people with bipolar disorders. So far, no complete *in silico* study has been conducted before that has predicted the nsSNPs in CAMKK2; to this end, the current study explores the deleterious SNPs that are associated with multiple diseases.

## 2. Materials and Methods

### 2.1. Sequence Retrieval

The protein sequence of CAMKK2 was obtained from UniProt [8] (https://www.uniprot.org/; UniProtKB: Q96RR4 (KKCC2_HUMAN)), and the structure was obtained from RCSB PDB (PDB ID: 2ZV2). This was followed by querying the NCBI dbSNP [9] to obtain the human CAMKK2 SNPs that broadly classify the SNPs based on their genomic locations (https://www.ncbi.nlm.nih.gov/snp/). dbSNP retrieved a total of 435 missense SNPs out of a total of 15,182 SNPs; hence, we picked only the missense SNPs for further analysis.

### 2.2. Prediction of nsSNPs Based on Sequence Homology

The missense SNPs were first functionally annotated using sequence-based tools, namely, SNPNexus (SIFT and PolyPhen) [10], PROVEAN [11], and Mutation Assessor [12]. SIFT and PolyPhen are built-in tools of SNPNexus which label the SNPs as probably damaging or benign based on the scores predicted. The tools are accessible at https://www.snp-nexus.org/v4/. PROVEAN (Protein Variation Effect Analyzer) is a sequence homology-based tool that runs the blast search on the query sequence to determine if the particular mutation is damaging or neutral. The cutoff threshold of PROVEAN is -2.5. The Mutation Assessor scores based on evolutionary conservation analysis that determines how conserved is the mutated residue, and based on that, it predicts the pathogenicity of a particular variant.

### 2.3. Prediction of nsSNPs Based on Consensus

The second group of tools are based on consensus methods, namely, Meta-SNP [13], SNPs&GO [14], and PredictSNP [15]. Meta-SNP combines a total of four predictors, namely, SNAP (Screening for Non-Acceptable Polymorphism) [16], SIFT (Sorting Intolerant from Tolerant), PANTHER (Protein Analysis through Evolutionary Relationships) [17], and PHD-SNP (Predictor of Human Deleterious SNP) (https://snps.biofold.org/meta-snp/). PredictSNP pools seven tools, namely, nsSNPAnalyzer [18], PolyPhen (Polymorphism Phenotyping), SNAP, MAPP (Multivariate Analysis of Protein Polymorphism), PHD-SNP, SIFT, and consensus PredictSNP (https://loschmidt.chemi.muni.cz/predictsnp/). The tool SNPs&GO has sequence- and function-derived features which include evolutionary conservation analysis and features derived from GO terms (https://snps-and-go.biocomp.unibo.it/snps-and-go/).

### 2.4. Prediction Based on Protein Sequence or Structure

The tools that fall in this category are mCSM [19], structure-based stability change prediction (STRUM) [20], and site-directed mutator (SDM) [21]. These three predictors either use sequence information or structural information to predict the protein stability changes upon mutation. These tools calculate the difference of the Gibbs free energy DDG value to determine the stability and instability of the protein structure upon mutation.

A SNP is categorized as high risk if it is predicted as deleterious from 7 out of these 9 tools to avoid any biases in the results.

### 2.5. Analysis of Evolutionary Conserved Residues

To analyze if the variants are occurring at evolutionary conserved residues, we have utilized the ConSurf webserver [22] available at http://consurf.tau.ac.il/2016/. The tool runs the sequence homology analysis via BLAST to generate the conservation-profile-labelled conservation scores. The scores ranging from 1 to 4 are labelled as variable, those ranging from 5 to 6 are labelled as intermediate, and those ranging from 7 to 9 are labelled as conserved. The tool defines the structurally and functionally important residues by identifying its location in the structure as buried or exposed.

### 2.6. Predicting Disease-Related Mutations Using MutPred

To predict the disease pathogenicity associated with the missense variants, the MutPred predictor was utilized [23]. MutPred takes the output of three tools, namely, Psi-BLAST, SIFT, and PFAM. The tool is accessible at http://mutpred.mutdb.org/. Further, the tool also combines three more structural disorder algorithms including TMHMM, MARCOIL, and DisProt. This combination will lead to a more confident prediction.

### 2.7. Protein Structure Modelling

The modelled 3D structure of wildtype CAMKK2 was deposited in RCSB PDB, and we have downloaded the PDB structure (PDB ID: 2ZV2) which covers the modelled residues from 158 to -448. As the full tertiary structure is not known, the I-TASSER homology modelling tool [24] was used to predict the full 3D structure of CAMKK2. This is a fully automated tool that uses threading and *ab initio* methods to determine the structure. Both the wildtype and mutant structures were generated using I-TASSER. The quality of the predicted structures was evaluated by ERRAT [25] to determine the structure quality of the wildtype and mutant models. Next, the wildtype and mutant structures were superimposed using TM-Align [26] to identify the location of mutations on the structure. It generates the superimposed structures along with the computed RMSD which determines how deviated the mutant models are from the wildtype. The higher the value, the more deviated the models are; hence, they are expected to cause more structural and functional damage.

### 2.8. Pathway Enrichment Analysis and Molecular Docking of CAMKK2

To determine the functional binding partners of CAMKK2, the STRING database [27] was used. The threshold for generating the network was set to 0.7. Further, the protein-protein docking studies were carried out using the ClusPro webserver [28] (https://cluspro.org/login.php) by using the default settings. The server uses the Fast Fourier Transform Correlation approach hence making it a flexible approach for docking. Further, ClusPro generates clusters of docked poses using the greedy approach that first rotates the ligand in 70,000 different angles, and based on the scores generated, 1000 poses were selected. On querying, the server generated 10 different docked poses which were ordered based on the energy and the cluster size.

## 3. Results

### 3.1. nsSNPs in CAMKK2

A total of 430 missense SNPs were downloaded from dbSNP; out of these, SNPNexus predicted 197 SNPs as possibly damaging to the protein. SNPNexus combines SIFT and PolyPhen as a built-in tools and takes an average voting to label a SNP as damaging or neutral. These SNPs were further subjected to other sequence homology-based tools.

First, the PROVEAN predicted 147 variants as deleterious, while the others are predicted as neutral. Only 8 mutations were predicted as highly damaging by Mutation Assessor. From the next group of tools which are based on consensus-based predictions, firstly, the Meta-SNP predicted 92 substitutions, secondly, the PredictSNP predicted 98 variants as diseased, and lastly, the predictions made from SNPs&GO shortened the list to 55 mutations as diseased. The last category of tools falls in protein sequence- and structure-based predictions for which we have used the STRUM, mCSM, and SDM online servers. STRUM predicted 99 variants as destabilizing, while mCSM and SDM predicted 18 as highly destabilizing.

Per category, we have selected 2 out of 3 tools as significant predictors, which makes 6 out of 9 tools on the whole as significant and high-confidence predictors. The combined results from functional annotation tools are tabulated in Table 1 and Table 2, while detailed predictions of the SNPs for each tool are tabulated in Supplementary File Tables S1–S5.

### 3.2. Evolutionary Conservation Analysis Using ConSurf

The evolutionary conservation analysis carried out by ConSurf has categorized the conservation as variable, intermediate, and conserved, with conserved categorized as buried; hence, it has a structural role or is exposed. Therefore, it has the functional role in the protein. The server predicted 105 total residues that occur at the surface of the protein and also possess a functional role in the protein, while 47 residues are conserved, buried, and have a structural role in the protein. The detailed results are tabulated in supplementary Table S6.

### 3.3. Predicting Disease-Associated Mutations Using MutPred

To interpret if the deleterious SNPs predicted above are also disease causing and whether there is any pathogenicity associated with them, we have utilized MutPred. The tool predicts the molecular process associated with mutants like altered disordered interface, gain or loss of catalytic sites, alterations of transmembrane helices, and posttranslational modifications. The *p* value = 0.05 and the *g* value > 0.75 are considered as significant. The mutants R311C, V195A, V249M, W445C, and F366Y have the highest MutPred scores as predicted. The detailed results are tabulated in Table S7.

### 3.4. Protein Structure Prediction and Molecular Docking

The deposited 3D structure of CAMKK2 has modelled residues from 158 to 448; therefore, we predicted the tertiary structure using the I-TASSER homology modelling tool that covers the full protein sequence. The mutant models were also generated using PyMOL further, and TM-Align was used to compute the structural similarity between wildtype and mutant models by generating the RMSD scores. The mutants R311C, V195A, V249M, W445C, and F366Y showed the highest deviation of 1.98, 1.65, 1.52, 1.78, and 1.34 Å, respectively. Next, the quality of the predicted structures was checked with ERRAT. The following quality factors were generated: for wildtype, 79.56; for mutant F366Y, 72.95; for R311C, 72.65; for V195A, 72.72; for V249M, 73.25; for W445C, 73.24; and for P389T, 78.78.

**Figure 1:**
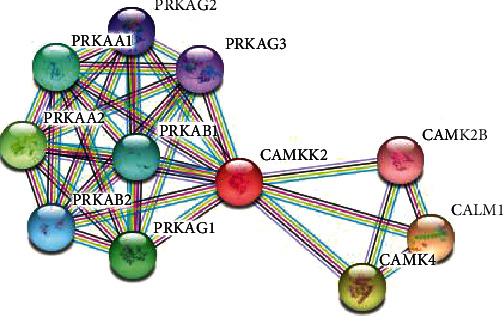
The protein network generated from the STRING database.

**Figure 2:**
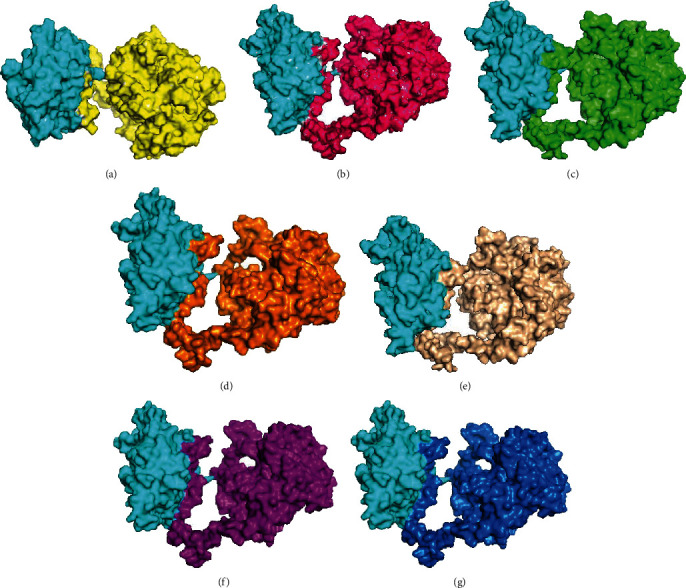
The buried surface area (BSA) for the protein-bound complex. (a) The CAMKK2 wildtype with PRKAG1. (b) The V195A (CAMKK2) variant with PRKAG1. (c) The V249M (CAMKK2) variant with PRKAG1. (d) The R311C (CAMKK2) variant with PRKAG1. (e) The F366T (CAMKK2) variant with PRKAG1. (f) The P389T (CAMKK2) variant with PRKAG1. (g) The W445C (CAMKK2) variant with PRKAG1.

**Figure 3:**
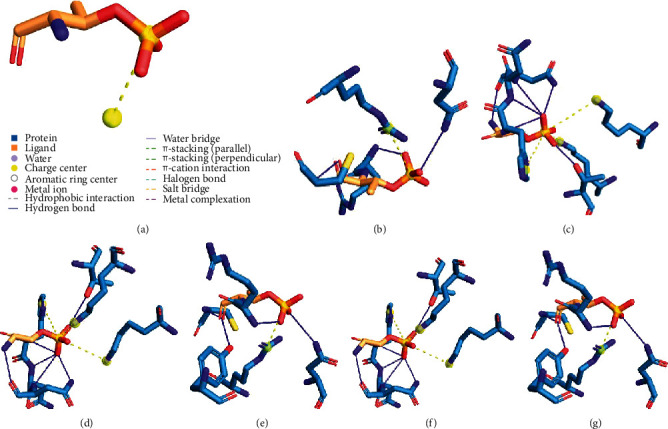
The interactive residues of the two interacting proteins in the wildtype model and mutant models. (a) The CAMKK2 wildtype with PRKAG1. (b) The R311C (CAMKK2) variant with PRKAG1. (c) The P389T (CAMKK2) variant with PRKAG1. (d) The W445C (CAMKK2) variant with PRKAG1. (e) The F366T (CAMKK2) variant with PRKAG1. (f) The V195A (CAMKK2) variant with PRKAG1. (g) The V249M (CAMKK2) variant with PRKAG1.

The results from STRING showed that CAMKK2 binds with PRKAG1, and it also activates and catalyzes the protein (Figure 1). In addition to that, the phosphorylation which is a posttranslational modification is also carried out by CAMKK2 to PRKAG1. So, our next task was to determine the binding affinities between the two proteins. For this purpose, we have utilized the ClusPro webserver. The wildtype models and the mutant models of CAMKK2 (PDB ID: 2ZV2) with PRKAG1 (PDB ID: 4CFE) were docked. To determine if the mutations are affecting the interactions of two proteins, we have calculated the buried surface area (BSA) which we considered as the measure of the strength of the bound complex. The difference of BSA will determine if the mutations are actually disturbing the protein-protein interactions. The protein complex with highest BSA is expected to be more stable as observed in the wildtype protein-protein docked model. The decrease in BSA clearly shows that the mutations are affecting the protein-protein interactions between CAMKK2 and PRKAG1 which is necessary for the signaling cascade.  Table 3 and Figure 2 show the computed buried surface area of the docked complexes of the wildtype and mutant models. Further, the interacting residues between CAMKK2 and PRKAG1 both in wildtype and mutant models were generated via the Protein-Ligand Interaction Profiler (PLIP) [29] accessible at https://projects.biotec.tu-dresden.de/plip-web/plip. The tool predicts the binding pocket which indicates that the different sets of residues are interacting with the wildtype docked model as compared to the mutant docked complexes (Figure 3). The details are listed in supplementary Table S8.

## 4. Discussion

This study carried out an in-depth analysis on the nsSNPs in the CAMKK2 protein by applying a combination of bioinformatics tools. First, the SNPs were functionally annotated using 9 different tools from 3 different categories, namely, sequence homology-based, consensus–sequence-based, and sequence–structure-based tools. This give us 14 mutations that are predicted as high risk from the abovementioned tools. The SNPs that were labelled as damaging by the functional annotation tools were further passed to evolutionary conservation analysis to identify if these variants are occurring at evolutionary conserved residues of the protein. ConSurf combines evolutionary information with the solvent accessibilities to identify conserved-structural and conserved-functional residues. From the conservation profile generated, we have focused only on those 13 mutations which were labelled as damaging from functional annotation. Next, the structural analysis of these 13 mutations were carried out. The wildtype model was generated using I-TASSER, while the mutant models were created from PyMOL by altering the sequence at the mutation position. Following this, the TM scores and RMSDs were computed to measure the distance of *α*-carbon backbones of wild type and mutant models. The higher the RMSD, the greater is the deviation of the mutant model from the wild type. The six variants V195A, V249M, R311C, F366Y, P389T, and W445C showed the maximum deviation from the wildtype protein.

Based on the higher values, we have finally selected these 5 mutations for docking studies. First, the wildtype models of CAMKK2 and PRKAG1 were docked using ClusPro, then the BSA was computed. Next, the six mutant models of CAMKK2 were docked with the wildtype PRKAG1, and BSA was computed for these docked complexes. As BSA was considered as the measure of strength of the protein bound complex, hence, the difference can shed light on the effect of mutations on the protein-protein interactions. Subsequently, these six mutations were analyzed with the HOPE webserver [30], which predicts that all these substitutions are occurring at the catalytic site of the protein kinase domain which is evolutionary conserved and regulates several cellular processes including apoptosis, cell differentiation, and proliferation. The W445C variant mutates tryptophan to cysteine, which introduces a size difference as tryptophan is bigger in size than cysteine; this can create a space in the core of the protein. Also, the wildtype makes a hydrogen bond with arginine at position 286, which cysteine might not make. The mutation might affect the interaction and thereby disturb signal transfer from the binding domain to the activity domain.

The V195A substitution replaces valine with alanine; valine is more hydrophobic then a mutant residue. Although, the mutated residue is not in direct contact with any ligand, the mutation is expected to affect the local stability of the protein which in turn could affect the ligand contacts made by one of the neighboring residues. The altered mechanisms with this variant as predicted from MutPred are gain of phosphorylation at the Y190 residue and altered DNA binding sites.

In the F366Y substitution, the hydrophobicity of the wildtype and mutant residue differs. The mutation will cause loss of hydrophobic interactions on the surface of the protein. The variant results in loss of relative solvent accessibility and loss of acetylation at K369 which affect the binding of CAMKK2 with other proteins or ligands. In V249M, the wildtype binds with the 34U ligand. The difference in properties between wildtype and mutated residue can clearly cause loss of interactions with the ligand. Also, it causes gain of allosteric site at H245 which clearly affects the binding with the ligand.

**Figure 4:**
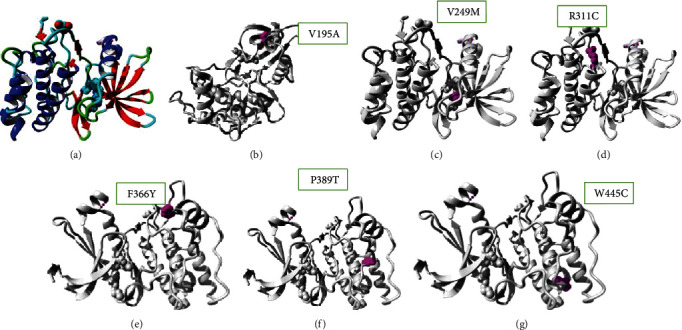
(a) Wildtype model of the CAMKK2 protein structure. (b) The location of the V195A mutant residue. (c) The V249M mutation. (d) The R311C mutation. (e) The F366T variant. (f) The P389T variant. (g) The P389T variant.

In the R311C variant, the wildtype residue forms a hydrogen bond with asparagine at positions 335 and 346. The size difference of the two residues will disrupt the position; hence, the mutated residue will not make the same hydrogen bond as the wildtype residue was making. The mutation also causes the gain of a catalytic site and an altered metal-binding site which disrupts the interaction at this position. In addition to that, the wildtype residue forms a salt bridge with aspartic acid at position 372. The difference in charge will disturb the ionic interaction that leads to the loss of interactions. Also, as predicted, the mutated residue is located very close to the active site, and the mutation at this position will affect the local structure surrounding the active site, hence affecting the function of the protein. The P389T substitution will result in the loss of a loop in the protein structure as proline is a rigid amino acid which introduces a special backbone conformation which might be lost by this mutation. The structure location of mutations in the protein structure predicted by HOPE is shown in Figure 4.

## 5. Conclusion

The current study is based on *in silico* bioinformatics analysis that combines sequence- and structure-based predictions to mark a SNP as damaging or neutral. The predicted six mutations V195A, V249M, R311C, F366Y, P389T, and W445C will create an evident reduction in the kinase activity which is necessary to regulate various cellular processes including cell differentiation and apoptosis. The variant R311C is already experimentally predicted in one of the studies reported in the past as mentioned in the Introduction, which further supports our methodology and provides our prediction with a more confident score. The other five nsSNPs can further be experimentally analyzed to depict more accurately their role in CAMKK2.

## Figures and Tables

**Table 1:** Deleterious SNPs predicted from homology-based tools, consensus-based tools, and MutPred.

Homology-based tools					Consensus-based methods				MutPred
Mutation	SNPNexus	PROVEAN	Mutation Accessor	Prediction	Meta-SNP	PredictSNP	SNPs&GO	Prediction	MutPred
E465H	Deleterious	Deleterious	Deleterious	*Deleterious*	Deleterious	Deleterious	Deleterious	*Deleterious*	*Neutral*
R508C	Deleterious	Deleterious	Deleterious	*Deleterious*	Deleterious	Deleterious	Deleterious	*Deleterious*	*Neutral*
R504W	Deleterious	Deleterious	Deleterious	*Deleterious*	Deleterious	Deleterious	Deleterious	*Deleterious*	*Neutral*
C461S	Deleterious	Deleterious	Deleterious	*Deleterious*	Deleterious	Deleterious	Deleterious	*Deleterious*	*Neutral*
D458H	Deleterious	Deleterious	Deleterious	*Deleterious*	Deleterious	Neutral	Neutral	*Deleterious*	*Neutral*
D458L	Deleterious	Deleterious	Deleterious	*Deleterious*	Deleterious	Deleterious	Deleterious	*Deleterious*	*Neutral*
P453Q	Deleterious	Deleterious	Neutral	*Deleterious*	Deleterious	Neutral	Deleterious	*Deleterious*	*Neutral*
P453C	Deleterious	Deleterious	Neutral	*Deleterious*	Deleterious	Neutral	Deleterious	*Deleterious*	*Neutral*
W445C	Deleterious	Deleterious	Neutral	*Deleterious*	Deleterious	Deleterious	Neutral	*Deleterious*	*Neutral*
H443Y	Deleterious	Deleterious	Neutral	*Deleterious*	Deleterious	Deleterious	Deleterious	*Deleterious*	*Neutral*
A182I	Deleterious	Deleterious	Deleterious	*Deleterious*	Deleterious	Deleterious	Deleterious	*Deleterious*	*Neutral*
P438R	Deleterious	Deleterious	Neutral	*Deleterious*	Neutral	Neutral	Neutral	*Neutral*	*Neutral*
M193T	Deleterious	Deleterious	Deleterious	*Deleterious*	Deleterious	Deleterious	Deleterious	*Deleterious*	*Neutral*
I435A	Deleterious	Deleterious	Neutral	*Deleterious*	Deleterious	Neutral	Deleterious	*Deleterious*	*Diseased*
V195A	Deleterious	Deleterious	Deleterious	*Deleterious*	Deleterious	Neutral	Deleterious	*Deleterious*	*Diseased*
R217W	Deleterious	Deleterious	Deleterious	*Deleterious*	Deleterious	Deleterious	Neutral	*Deleterious*	*Neutral*
P389T	Deleterious	Deleterious	Deleterious	*Deleterious*	Deleterious	Deleterious	Deleterious	*Deleterious*	*Diseased*
F366Y	Deleterious	Deleterious	Neutral	*Deleterious*	Neutral	Neutral	Neutral	*Neutral*	*Diseased*
L419H	Deleterious	Deleterious	Neutral	*Deleterious*	Neutral	Deleterious	Deleterious	*Deleterious*	*Diseases*
R492C	Deleterious	Deleterious	Neutral	*Deleterious*	Deleterious	Deleterious	Deleterious	*Deleterious*	*Neutral*
V249M	Deleterious	Deleterious	Deleterious	*Deleterious*	Deleterious	Deleterious	Deleterious	*Deleterious*	*Diseased*
V487M	Deleterious	Deleterious	Neutral	*Deleterious*	Deleterious	Deleterious	Deleterious	*Deleterious*	*Neutral*
Y183C	Deleterious	Deleterious	Deleterious	*Deleterious*	Deleterious	Neutral	Neutral	*Neutral*	*Diseased*
N460K	Deleterious	Deleterious	Deleterious	*Deleterious*	Deleterious	Deleterious	Deleterious	*Deleterious*	*Neutral*
L318A	Deleterious	Deleterious	Neutral	*Deleterious*	Deleterious	Deleterious	Deleterious	*Deleterious*	*Diseased*
M265T	Deleterious	Deleterious	Neutral	*Deleterious*	Neutral	Neutral	Neutral	*Neutral*	*Diseased*
M265D	Deleterious	Deleterious	Deleterious	*Deleterious*	Deleterious	Deleterious	Deleterious	*Deleterious*	*Diseased*
R311C	Deleterious	Deleterious	Deleterious	*Deleterious*	Deleterious	Deleterious	Deleterious	*Deleterious*	*Diseased*
V233L	Deleterious	Deleterious	Neutral	*Deleterious*	Neutral	Neutral	Neutral	*Neutral*	*Neutral*
P444L	Deleterious	Deleterious	Neutral	*Deleterious*	Deleterious	Deleterious	Deleterious	*Deleterious*	*Neutral*
E432A	Deleterious	Deleterious	Deleterious	*Deleterious*	Deleterious	Deleterious	Deleterious	*Deleterious*	*Neutral*
V275T	Deleterious	Deleterious	Deleterious	*Deleterious*	Neutral	Neutral	Neutral	*Neutral*	*Diseased*
P389T	Deleterious	Deleterious	Deleterious	*Deleterious*	Neutral	Deleterious	Deleterious	*Deleterious*	*Diseased*

**Table 2:** Deleterious SNPs predicted from protein stability analysis and evolutionary conservation analysis.

SNP	Mutation	Evolutionary conservation	DDG and effect
rs1291358851	F366Y	Highly conserved, exposed	Destabilizing
rs1305210574	V195A	Highly conserved, exposed (f)	Highly destabilizing
rs200059037	V249M	Highly conserved, buried (structural)	Highly destabilizing
rs1307905721	R311C	Highly conserved, exposed (f)	Highly destabilizing
rs1219582970	W445C	Highly conserved, buried (structural)	Highly destabilizing
rs746740827	Y183C	Conserved, exposed	Highly destabilizing
rs746740827	Y183T	Conserved, exposed	Highly destabilizing
rs767392357	L318A	Conserved, buried	Highly destabilizing
rs751909766	I435A	Conserved, buried	Highly destabilizing
rs1459757320	L419H	Conserved, buried	Highly destabilizing
rs769333324	P389T	Conserved, exposed (functional)	Highly destabilizing
rs1354124033	V275T	Conserved, buried	Highly destabilizing
rs1275152562	M265T	Conserved, buried	Highly destabilizing
rs1275152562	M265D	Conserved, buried	Highly destabilizing

**Table 3:** Buried surface area of the wildtype bound complex and the mutant bound complex.

CAMKK2-PRKAG1-bound complex	Buried SA (*A*^2^)
Wildtype interactions	4450
V195A-PRKAG1	3204
V249M-PRKAG1	3201
R311C-PRKAG1	3227
F366Y-PRKAG1	3214
W445C-PRKAG1	3202
P389T-PRKAG1	3186

## Data Availability

All the data is available in the main manuscript and in the supplementary files.

## Supplementary Materials

Supplementary Materials Table S1 lists down the SNPs that are predicted from SNPNexus (SIFT and PolyPhen). Table S2 shows the deleterious SNPs predicted from the PROVEAN tool. Table S3 summarizes the results from the Mutation Assessor tool that has a function prediction category as medium, low, and high. Table S4 shows the combined SNPs that are predicted as damaging from Meta-SNP, PredictSNP, and SNPs&GO. Table S5 has the list of SNPs that are predicted as destabilizing the protein structure and function. Table S6 shows the SNPs that are predicted as evolutionary conserved from the ConSurf tool. Table S7 shows the disease causing mutations from MutPred. Table S8 shows the interacting residues generated from PLIP for wildtype and mutant docked complexes.
